# Editorial: Nutritional Intervention for the Intestinal Health of Young Monogastric Animals

**DOI:** 10.3389/fvets.2021.668563

**Published:** 2021-03-18

**Authors:** Rajesh Jha, Sung Woo Kim

**Affiliations:** ^1^Department of Human Nutrition, Food and Animal Sciences, University of Hawaii at Manoa, Honolulu, HI, United States; ^2^Department of Animal Science, North Carolina State University, Raleigh, NC, United States

**Keywords:** antimicrobial resistance, gut health, prebiotics, probiotics, pig, poultry

## Introduction

Poultry and pig production have increased at a faster rate than any other livestock production globally (1). Among others, nutritionally balanced-feeding programs, along with antibiotic growth promoters (AGP) in feeds, played a significant role in achieving this success (2, 3). The animal industry, however, aims to redefine its nutrition program to grow safe and quality meat in the light of public health concerns due to the use of AGP in diets (1). Maintenance or improvement of intestinal health is essential for optimum growth, better feed efficiency, and the overall health of pigs and poultry (4–6). Keeping a healthy intestine is also critically important for nutrient digestion and utilization, thereby ensuring better growth performance of pigs and poultry (7–9).

Intestinal health covers efficient nutrient utilization, macro- and micro-structural integrity of the gut, the stability of the microbiota, and the status of the immune system (4, 10, 11). Moreover, intestinal health is a complex field combining the nutrition, microbiology, immunology, and physiology of animals. Challenges in intestinal health directly influence nutrient digestion and absorption (4, 12, 13), which in turn reduces feed efficiency and increases susceptibility to enteric diseases (14, 15).

Recent regulatory changes on the use of AGP and selected feedstuffs have challenged the optimal growth and health of modern pigs and poultry, that have been extensively selected for growth efficiency and lean gain. Highly lean and fast-growing pigs and poultry highlight the need for a better understanding of the gut function and overall gut health. Understanding and improving the intestinal health of animals is a key essential trend needed for the success of animal production in this era of AGP free production (1, 4, 16). This Research Topic eBook covers nutritional aspects of improving the intestinal health of monogastric animals, including current challenges and potential solutions. The papers have been presented under two sections: (1) Importance and understanding intestinal health of monogastric animals and (2) Nutritional intervention for intestinal health.

## Importance and Understanding The Intestinal Health of Young Monogastric Animals

It is well-established that effective modulation of the gut health parameters depends not only on feedstuffs but also on the methods and timing of the nutrients available to host animals. Furthermore, early growth and development of GIT are of critical importance in enhancing nutrient utilization and optimizing the growth of poultry. Early nutrition programming using both *in ovo* and post-hatch feeding has been used as a means to modulate the early growth and development of GIT and has been found to be an effective strategy [(17); (Jha, Singh et al.)]. Similarly, the weaning phase of pigs is an incredibly stressful period as it causes morphological and functional changes in the gut and induces post-weaning growth depression. Different nutritional strategies, including the addition of functional feed additives in the weaner pig's diet have been proposed to minimize these effects (Zheng et al.). Similarly, different nutrients and feed additives have been used to optimize the gastrointestinal integrity and immune system of young animals (Adedokun and Olojede). However, gut microbiota plays a significant role in managing the gut environment by producing fermentation metabolites and influencing nutrient utilization pathways (18). Thus, it is important to understand the gut microbial ecology in-depth, including their taxonomic composition and biochemical functions. However, the gut microbiota is primarily influenced by diet, age, species, and location in the digestive tract (5, 18, 19). Different techniques have been used to characterize the gut microbiota, but those have different strengths and limitations. Modern techniques like 16S rRNA based next-generation sequencing and others are powerful tools to investigate the biological and ecological roles of the gut microbiota [(19); (Shang et al.)]. In the commercial animal production system, different nutritional and environmental stresses and pathological factors create oxidative stress in animals, leading to imbalances in the intestinal homeostasis due to the generation of reactive oxygen species (ROS) and reactive nitrogen species. It can be mitigated by supplementing exogenous vitamins, antioxidants, and plant extracts that have antioxidant properties that scavenge ROS [(9); (Mishra and Jha)]. Thus, it is crucial to understand the involvement of oxidative stress in the gastrointestinal functionality of animals and the potential intervention strategies available to maintain redox balance in the GIT.

## Nutritional Intervention for Intestinal Health

It is not only the type of feedstuffs, but also their forms that have been found to affect gut health and function. A finer feed particle size enables optimal nutrient utilization and enhances animal performance due to increased surface area, allowing for better contact with digestive enzymes. Moreover, adequate diminution of feedstuffs is beneficial to feed manufacturing processes such as mixing and hydrothermal treatments, including pelleting, extrusion, and expansion. Thus, feed processing techniques, along with the type of feedstuffs, need to be considered when formulating diets for animals considering their impact on intestinal health (Kiarie and Mills).

As various feedstuffs, their components, and feed additives behave and function differently in the GIT of animals, different feeding strategies have been tested, with some success, to improve intestinal health and functionality. Furthermore, there is also a need to evaluate potential alternatives to AGPs in animal diets in the post-antibiotic era [(3); (Yang et al.)]. As potential alternatives to AGPs, different dietary fibers (DF), prebiotics, probiotics, post-biotics, enzymes, and others have been evaluated and found to have promising outcomes (Zheng et al.). Although DF are not well-digested and are often considered as anti-nutritional factors in monogastric animals, as it reduces nutrient utilization (20), it has been widely used in recent years to modulate the intestinal environment (4). DFs are fermented in the intestine and become short-chain fatty acids, stimulating the growth of health-promoting gut bacteria, and boosting the immune system [(21); (Jha, Fouhse et al.)]. In addition, specific nutrients such as functional amino acids like arginine, cysteine, glutamine, or glutamate, may enhance intestinal mucosa immunity, reduce oxidative damage, stimulate proliferation of enterocytes, and enhance the gut barrier function of weaned pigs (Xiong et al.). Amino acids, which are major nutrients for monogastric animals, are not only obligatory for maintaining the intestinal mucosal mass and integrity, but also for supporting the growth of microorganisms in the gut. Dietary amino acids are the major fuel of the small intestinal mucosa. Particularly, glutamate, glutamine, and aspartate are the primary oxidative fuel of the intestine (Yang and Liao). Trace minerals like copper, zinc, iron, and manganese have also been found to influence gut health parameters (Shannon and Hill). For example, pharmacological concentrations of copper have been shown to enhance growth, while high concentrations of zinc fed to newly weaned nursery pigs reduced the incidence of diarrhea from the proliferation of enterotoxigenic *Escherichia coli* and *Clostridium* and improved gut morphology. As a potential alternative to AGPs, prebiotics including mannan oligosaccharides, b-glucans, and fructans, are gaining more attention to be used in monogastric feeding program as prebiotics have been found to modulate microbial communities and regulate the production of cytokines and antibodies, improving gut development and the overall health of animals (Teng and Kim). Similar to prebiotics, different probiotics alone or in combination with other additives like enzymes have also been tried to improve intestinal health and nutrient utilization (3, 22). Duarte et al. evaluated the symbiotic effect of prebiotic (*Bacillus* sp.) and xylanase enzyme in *E. coli* F18+ challenged weaned pigs. The study found that the feed additives were able to mitigate the negative effects of *E. coli* F18+ infection in pigs fed an antibiotic-free diet and enhanced the growth performance by reducing diarrhea, boosting immune response, and managing oxidative stress in the jejunum. In addition, Ma et al. found that synbiotic supplementation in the maternal diet positively affects the gut health of piglets, including improving nutrient metabolism, reducing oxidative stress, and improving intestinal barrier permeability function. Similarly, Grosu et al. used grapeseed meal (GSM) with bioactive compounds (such as polyphenols, PUFA, DF, minerals, etc.) in pig diets. They found that the grapeseed meal had a selective modulatory effect on several bacterial genera in the colon of pigs challenged with dextran sodium sulfate, as a model for inflammatory bowel diseases, suggesting that the GSM can be used as a potential anti-inflammatory additive in weaned piglets.

In conclusion, there are different dietary components and feed additives that can be used to modulate the intestinal health and functions of young monogastric animals. It can be a tool for nutritionists to develop a feeding program in the post-antibiotic era. However, types, forms, and dose levels of these dietary components and additives need to be considered to obtain optimum benefits.

## Author Contributions

All authors listed have made a substantial, direct and intellectual contribution to the work, and approved it for publication.

## Conflict of Interest

The authors declare that the research was conducted in the absence of any commercial or financial relationships that could be construed as a potential conflict of interest.
